# *Plasmodium falciparum *clearance with artemisinin-based combination therapy (ACT) in patients with glucose-6-phosphate dehydrogenase deficiency in Mali

**DOI:** 10.1186/1475-2875-9-332

**Published:** 2010-11-21

**Authors:** Abdoulaye K Kone, Issaka Sagara, Mahamadou A Thera, Alassane Dicko, Aldiouma Guindo, Seidina Diakite, Joseph Kurantsin-Mills, Abdoulaye Djimde, Asikiya Walcourt, Ogabara Doumbo

**Affiliations:** 1 Malaria Research and Training Center, Faculty of Medicine, Pharmacy and Odonto-stomatology, University of Bamako, P. O. Box 1805 Bamako, Mali; 2 Department of Physiology & Biophysics, Howard University College of Medicine, 520 W Street, Rm 413, Seely G. Mudd Building, NW, Washington, DC 20059, USA; 3 Departments of Medicine, Pharmacology & Physiology, The George Washington University Medical Center, Washington, DC 20037, USA

## Abstract

**Background:**

Artemisinin-based combination therapy (ACT) is currently the most effective medicine for the treatment of uncomplicated malaria. Artemisinin has previously been shown to increase the clearance of *Plasmodium falciparum *in malaria patients with haemoglobin E trait, but it did not increase parasite inhibition in an *in vitro *study using haemoglobin AS erythrocytes. The current study describes the efficacy of artemisinin derivatives on *P. falciparum *clearance in patients with glucose-6-phosphate dehydrogenase deficiency (G6PD), a haemoglobin enzyme deficiency, not yet studied in the same context, but nonetheless is a common in malaria endemic areas, associated with host protection against uncomplicated and severe malaria. The impact of G6PD deficiency on parasite clearance with ACT treatment was compared between G6PD-deficient patients and G6PD-normal group.

**Methods:**

Blood samples from children and adults participants (1 to 70 years old) with uncomplicated *P. falciparum *malaria residing in Kambila, Mali were analysed. Study participants were randomly assigned to receive either artemether-lumefantrine (Coartem^®^) or artesunate plus mefloquine (Artequin™). A restriction-fragment length polymorphism analysis of PCR-amplified DNA samples was used to identify the (*A-*) allele of the gene mutation responsible for G6PD deficiency (G6PD*A-). 470 blood samples were thus analysed and of these, DNA was extracted from 315 samples using the QIAamp kit for PCR to identify the G6PD*A- gene.

**Results:**

The DNA amplified from 315 samples using PCR showed that G6PD*A- deficiency was present in 56 participants (17.8%). The distribution of the specific deficiency was 1%, 7% and, 9.8% respectively for homozygous, hemizygous, and heterozygous genotypes. Before treatment, the median parasitaemia and other baseline characteristics (mean haemoglobin, sex and age groups) between G6PD deficiency (hemizygous, heterozygous, and homozygous) and G6PD-normal participants were comparable (p > 0.05). After treatment, parasite clearance did not change significantly whether the participants were G6PD deficient or G6PD normal on day 1 (OR = 1.3; CI = 0.70-2.47; p > 0.05) and on day 2 (OR = 0.859; CI = 0.097-7.61; p > 0.05).

**Conclusions:**

The presence of G6PD deficiency does not appear to significantly influence the clearance of *P. falciparum *in the treatment of uncomplicated malaria using ACT.

## Background

In a race to combat the ever increasing resistance of *Plasmodium falciparum *to older anti-malarial drugs, artemisinin, a natural product found in the leafy portions of *Artemisia annua *(qinghao) and its derivatives, have emerged as alternative drugs for the treatment of falciparum malaria [1]. Artemisinin derivatives are sesquiterpenoides with an endoperoxide, which is the essential component of the anti-malarial activity. With their structural distinction from all other anti-malarial, artemisinins have so far been shown to be effective against multidrug-resistant strains of *P. falciparum*. The use of artemisinin-based combination therapy (ACT) is associated with a rapid clearance of the parasite and a low probability of drug-resistant parasite emergence [2]. ACT is currently recommended by World Health Organization (WHO) for treating uncomplicated falciparum malaria. Several reports evaluating the efficacy of ACT have confirmed its efficacy and safety. Studies by Falade *et al *in Nigeria showed a 28-day cure rate of about 95% with artemether-lumefantrine (AL) [3] and 93% for artesunate-amodiaquine (ASAQ) [4]. Karema *et al *also reported day 28 cure rates of 95.2% and 92.0% for dihydroartemisinin/piperaquine (Artekin) and ASAQ, respectively, in Rwanda [5].

Studies have also shown *P. falciparum *susceptibility to anti-malarial drugs to correlate with abnormal haemoglobins. A clinical study in Thailand first suggested that haemoglobin E trait (with characteristically increased oxidative activity due to excess α-globin chains) interacts with artemisinin derivatives to enhance *P. falciparum *clearance in malaria patients with haemoglobin E trait compared to patients treated with other anti-malarials [6]. Another study found a reduced chloroquine and artemisinin efficacy against *P. falciparum *in α-thalassemia [7]. Other studies found no differences in parasite susceptibility to chloroquine in Hb AS and Hb AA red blood cells *in vitro *[8]. More recently, an *in vitro *study has demonstrated no evidence of elevated artemisinin activity on *P. falciparum *in haemoglobin AS erythrocytes [9]. These conflicting data on the activity of artemisinin on *P. falciparum *in abnormal haemoglobin carriers make it imperative to assess the efficacy of ACT in G6PD deficient patients in Mali, where this haemoglobinopathy is common. As far as the literature is concerned, no such studies have been done to study the efficacy of artemisinin against *P. falciparum *in G6PD deficient patients. G6PD variants correlates with historical distribution of malaria [10] and the mutant allele (*A-*) encoding G6PD with 10%-50% of abnormal enzyme activity is widespread in Africa [11]. The aim of this study was to determine the clearance of *P. falciparum *and treatment efficacy in uncomplicated malaria as assessed by WHO 28-day protocol using ACT. The study hypothesis was that treatment with ACT may induce faster parasite clearance in G6PD deficient patients compared to G6PD normal patients.

## Methods

### Setting and study design

The study participants were selected from a randomized study conducted in Kambila village. The study participants were randomized to receive artemisinin-based combination therapy artemether-lumefantrine (Coartem^®^) or artesunate plus mefloquine (Artequin™). The G6PD mutant gene was identified by nested PCR performed on stored blood samples (filter paper dot). The study hypothesis was based on fact that *P. falciparum *in G6PD deficient patients would clear faster after ACT initiation compared to normal patients. A nested case control design was used to compare G6PD deficient to G6PD-normal subjects with a ratio of one case for four controls.

### Study population

The study participants were selected from children and adults (1 to 70 years old) patients who came to the health centre for care during the study period. Only patients with uncomplicated malaria who gave their consent after explanation of study objectives and understood were enrolled into the study. Children were enrolled after obtaining a written parental or guardian fully informed consent. Blood samples were collected from both children and adult patients and were analysed for G6PD status, and parasite clearance since it has been shown that parasite clearance is faster in adults than children [12].

### Study site and period

Study was conducted during a malaria transmission season from August 2004 through January 2005, in Kambila, Mali. Kambila is a peri-urban village of Kati located about 25 km from Bamako with a population of approximately 1,500 inhabitants. Malaria in this area is hyper-endemic and transmission is highly seasonal. *P. falciparum *is the predominant infecting species, accounting for more than 95% of malaria cases [13]. Kambila is one of the Mali malaria research sites where malaria drug efficacy data are available, and a prevalence of as high as 17% G6PD deficiency has also been documented [14].

### Sample size

The sample size was computed based on 70% of parasite clearance observed 24-hours after artesunate/amodiaquine treatment initiation in a general population at Bougoula-Hameau, Sikasso site, a similar malaria hyper-endemic area of Mali. Assuming an error risk alpha set at 0.05, a power of 80%, for one case matched with four controls and fixing a detectable risk estimate at 3.0 (Odds ratio), we will need 55 samples in the cases group (G6PD deficient) and 220 in the control group (non G6PD deficient), rounded up to 56 cases and 259 controls.

### Inclusion and exclusion criteria

Participants were included if they fulfilled the following criteria: 1) weighed ≥ 10 kg; 2) had a *P. falciparum *parasite density of between 2,000 and 200,000/μl; 3) had an auxiliary temperature ≥ 37.5°C; 4) were a resident of the study site; and 5) could take medication orally.

Persons were excluded if they had symptoms or signs of severe malaria [15], had a serious disease, had an allergy to one or more study drugs, had used any component of the study drugs within 28 days of enrollment, or were pregnant (detected either clinically or with a urine ß-human chorionic gonadotropin test). Community permission and individual written informed consent were provided by a parent or guardian of all participating children, as described by Diallo *et al *[16]. Withdrawal criteria for the study included (a) protocol violation, (b) participant withdrawal of consent, and (c) development of serious adverse event.

### Ethics considerations

The protocol was reviewed and approved by the ethical committee of the Faculty of Medicine, Pharmacy and Odonto-Stomatology of the University of Bamako before starting the randomized clinical trial. Approval letter from these three Faculty ethical of committees was also obtained before conducting the molecular analysis for the G6PD deficiency study.

### Laboratory procedures

Giemsa-stained thick blood smears were read by experienced laboratory technicians who were blinded to treatment allocations. Parasite densities were calculated by counting the number of asexual parasites until 300 leukocytes were observed and then converting that to parasites per microlitre of blood, assuming an average leukocyte count of 7,500/μl. For quality control, 10% of the slides were selected at random and re-read by another lab technician who was unaware of the results of the first reading.

Two methods were used for DNA extraction on blood spotted filter paper:

(1). An initial extraction was done using methanol. If no DNA could be extracted from blood samples or if the results of the PCR were conflicting, DNA was extracted using the QIAamp kit [17]. (2). If after an initial extraction using methanol yielded no results, or if the results yielded were conflicting, the QIAamp kit [17] was used to extract DNA.

The (*A*_) allele of the gene responsible for G6PD deficiency was determined by restriction fragment length polymorphism analysis of PCR-amplified DNA samples. Under conditions intended to eliminate the risk of cross-contamination and with appropriate water-only negative controls, exon 4 of G6PD was amplified using a nested PCR protocol: 1 μg of genomic DNA was first amplified using primers 5'-GTCTTCTGGGTCAGGGAT-3' (forward) and 5'-GGAGAAAGCTCTCTCTCC-3' (reverse). Denaturation at 94°C for 2 min was followed by 45 cycles of denaturation at 94°C for 30 s, annealing at 60°C for 30 s, extension at 72°C for 60 s, and final extension at 72°C for 4 min. Nested amplification was performed using primers 5'-CCTGTTCCCTCTGCCACA-3' (forward) and 5'-GGGGGTCTCAAGAAGTAC-3' (reverse). Denaturation at 94°C for 2 min was followed by 35 cycles of denaturation at 94°C for 30 s, annealing at 60°C for 60 s, extension at 72°C for 30 s, and a final extension at 72°C for 4 min. Amplification products were recovered with separate pipettes in laboratory areas apart from the PCR-preparation bench and digested with the restriction endonuclease NlaIII [18] or its isoschizomer Hsp92II [19] to detect the G6PD*A mutation at nucleotide position 202. Complete cutting of PCR product identified hemizygous males and homozygous females and cutting of half of the product identified heterozygous females.

### Participants surveillance

Study participants were examined at 1, 2, 3, 7, 14, 21, and 28 days after enrollment or at any time if they did not feel well. A finger skin puncture was used to obtain blood for a thick blood smear and a filter paper dot (for future parasite DNA extraction) at each follow-up visit.

### Study endpoints

The primary study endpoint was the time to parasitaemia disappearance from day 1 after treatment initiation. Parasitaemia clearance was defined as the parasitaemia disappearance at day 1, day 2 and up to day 28 after treatment. Proportion of gametocytes carriers at day 3 was defined as the percent of subjects carrying gametocytes that day regardless of their gametocyte carriage on day 0. The efficacy of ACT was assessed as an adequate clinical and parasitological response at day 28 according to WHO 2003 guideline [20].

### Statistical analysis

Data were double-entered, validated using Microsoft Access (Microsoft Corporation, Washington, USA), and analysed with STATA version 10.0 (STATA Corporation, College Station, TX). Chi-square test or Fisher exact test were used to compare proportions between groups. Mann-Whitney test or Kruskal Wallis test were used as appropriate to assess differences of median parasitaemia between groups for continuous variables. Logistic regression was used to control confounding factors, the odds ratio and exact 95% CIs were calculated. A *P *value (two-sided) < 0.05 was considered as statistically significant.

## Results

The DNA amplified from 315 samples out of the 470 samples using PCR showed that G6PD*A- deficiency was present in 56 participants (17.8%). The distribution of the specific deficiency was 1%, 7% and, 9.8% respectively for homozygous, hemizygous, and heterozygous genotypes. The mean haemoglobin and the mean body temperature were compared and no difference was seen between G6PD deficient genotype and normal genotype (p > 0.05) (Table 1).

**Table 1:** Distribution of G6PD deficiency genotypes according to age groups, body temperature and hemoglobin level at enrollment

G6PD Genotypes	Temperature	Haemoglobin Hb/dl	Age groups				
	Mean°C (Range)	Mean (Range)	< 5 years	5-10 years	11-17 years	>17 years	Total Number
			n (%)	n (%)	n (%)	n (%)	n (%)
Homozygous	38.0 (0.8)	11.4 (1.6)	1 (0,9)	2 (1,3)	0 (0)	0 (0)	3 (1)
Hemizygous	38.5 (2.8)	11.2 (5.3)	5 (4.7)	13 (8.7)	3 (6,1)	1 (10)	22 (7)
Heterozygous	38.5 (2.3)	11.4 (8.0)	6 (5,6)	14(9,4)	9 (18,4)	2 (20)	31 (9,8)
Normal	38.5 (2.9)	11.2 (9.1)	95(88.8)	120(80.5)	37 (75.5)	7(70.0)	259 (82,2)
Total	38.5 (2.9)	11.2 (9.1)	107 (100)	149 (100)	49 (100)	10 (100)	315 (100)

Fisher exact test p = 0.2 for G6PD Genotypes within age groups.

ANOVA p ≥ 0.05 for G6PD Genotypes and body temperature mean or haemoglobin mean

The G6PD deficiency genotypes were distributed equally between age groups (p = 0.2). The risk estimate of *P. falciparum *clearance at day 1, for homozygous, hemizygous or heterozygous G6PD deficiency versus G6PD-normal was not significant (Table 2). When median parasitaemia at day 0 for the normal G6PD was compared to the G6PD non-normal in general, there was no difference (p = 0.1).

**Table 2:** Risk estimate of *P. falciparum *clearance at day 1, G6PD deficiency genotypes versus G6PD-normal

	n	OR (95% IC)	P
Genotypes of G6PD			
Homozygous	3	0.894 (0.079-10.12)	0.93
Hemizygous	22	0.842 (0.33-2.15)	0.72
Heterozygous	31	0.695 (0.303-1.59)	0.40
Normal	258	1 (Reference)	

**Figure 1:**
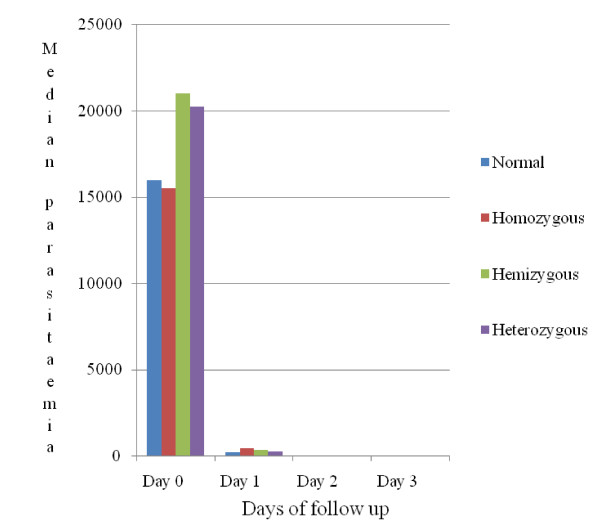
Clearance and density of *P. falciparum *from day 0-day 3, G6PD deficiency genotypes versus G6PD-normal.

From day 0 to day 3 the comparison of median parasite density and parasite clearance with respect to the different G6PD genotypes including the normal group didn't show any significant difference between groups (Figure 1).

Data were analysed by age groups (age groups as indicated in table 1) to assess age specific effect on parasite. The study results did not show significant differences between patients with G6PD deficiency compared to patients without the deficiency on day 1. The ACT efficacy treatment on day 28 was similar in the G6PD deficient group compared to the non-deficient group (Table 3). Gametocytes carriage at day 3 was not influenced by G6PD deficiency when the G6PD-normal group was compared to G6PD deficient group (Table 4).

**Table 3:** Association between G6PD deficiency and 28-day follow-up PCR corrected ACT efficacy

Efficacy	Hemizygous n (%)	Heterozygous n (%)	Homozygous n (%)	Normal n (%)	Total n (%)
Cured*	20 (100)	29 (96.6)	3 (100.0)	237 (98.02)	299 (98.03)
Failure cases	0 (0.0)	1 (3.33)	0 (0.0)	5 (1.98)	6 (1.97)

Total	20 (100)	30 (100)	3 (100)	252 (100)	305 (100)

*Adequate Clinical and Parasitological Response

Fisher exact test = 0.685

**Table 4:** Comparison of proportions of gametocytes carriers at day 3, G6PD deficient patients versus G6PD-normal patients

	Proportion of gametocytes carriers		
	
G6PD Status	Gametocytes (-)	Gametocytes (+)	Total
	
	n (%)	n (%)	n
Deficiency	54 (96.4)	2* (3.6)	56
Normal	252 (97.7)	6 (2.3)	258

Total	306 (97.5)	8 (2.5)	314

Fisher exact test p = 0.63

*The two cases occurred in heterozygous genotype deficiency

## Discussion

The purpose of this study was to determine the combined effect of G6PD deficiency and ACT treatment (artemether-lumefantrine and artesunate plus mefloquine) on parasite clearance in patients with uncomplicated falciparum malaria. Data were analysed by age groups to assess age-specific effect on parasite clearance in patients with G6PD deficiency, since studies have shown that parasite clearance was faster in adults than in children [12,21]. The current study shows no significant difference in parasite clearance between G6PD deficient patients compared to patients without the deficiency when adjusted for age effect.

The overall prevalence of G6PD deficiency was 17.8% is comparable to other authors finding reported elsewhere in Africa where the prevalence is from 0% to 25% [22]. The prevalence of 22.2% was reported in Congo [23] Genotype frequency was 9.8% (31/315), 7% (22/315), and 1% (3/315) for female heterozygous, male hemizygous and female homozygous respectively. The study findings are similar to those reported in other studies where they also observed genotype frequencies of 12% (36/303) for female heterozygous, 8% (25/303) for male hemizygous and < 1% (1/303) for homozygous females [24].

Parasite clearance between the different genotypes at day 0 was not statistically significant as median parasitaemia between abnormal and normal G6PD genotypes was not different. The study results have shown that hemizygous and heterozygous had a tendency of elevated median parasitaemia. Similar observation with geometric mean parasitaemia was also reported elsewhere [24].

A comparison of the median parasitaemia at day 0 between normal G6PD patients and G6PD deficient patients in general showed no difference (median parasite of 20212 parasites/μl versus 15975 parasites/μl respectively; p = 0.1). The study results did not confirm findings by Nkuo-Akenji et al., 2004 who showed a significant difference in the mean parasite density in G6PD enzyme deficient group compared to the G6PD normal group. That study found a mean parasite density of 3.7 (± 3.9, SD) for enzyme deficient individuals and 4.4 (± 5.0, SD) for enzyme active individuals (p < 0.05) [25].

The study results have shown that G6PD deficiency had no significant association with parasite clearance and median parasitaemia from day 0 to day 3 (Figure 1). The detectable risk estimate of three-fold parasite clearance used to calculate the sample size in this study may also be too high risk estimate, which may have resulted in a small simple size and, therefore, underpowered the study to detect any small differences in parasite clearance that may have been observed between deficient and non deficient G6PD patients on day 1 or day 2.

Other factors such as the molecular biology methods used to determine G6PD deficiency used in our study may also undermine the role of G6PD deficiency in parasite clearance. Enzyme assay appears to better reflect the biological effects of the G6PD enzyme more than genotyping. This was suggested in a recent study which showed that among female G6PD- deficient patients, but not male patients, whose genotype was also confirmed by enzymatic methods to have low enzyme activity as shown by enzyme assay were significantly protected from uncomplicated malaria, while patients with higher levels of enzyme activity were not protected against uncomplicated malaria [26].

No significant association between ACT efficacy and G6PD deficiency was seen in our data (Table 4). Although host immune response could play a role in treatment efficacy as other studies have demonstrated [27], the fast parasite clearance and treatment efficacy observed in this study regardless of the G6PD deficiency status is similar to what has been observed in other ACT trials in Mali [13,28]. This is indicative of the high efficacy rates in general of the ACT in Mali.

The possibility of reduced susceptibility of parasites to ACT in G6PD deficient patients could not also be ruled out since in a study with α-thalassemia patients, it was hypothesized that the infected variant erythrocytes could not accumulate as much drug as infected normal erythrocytes (7, 9). Furthermore, the authors had shown that the reduction in artemisinin accumulation in infected variant erythrocytes was due partly to competition with uninfected erythrocytes for the drug, and partly to the lower drug accumulation inside the parasite [7,29]. Therefore, a possible lower drug accumulation in parasites in G6PD deficient red cells could be a disadvantage to G6PD-deficient carriers. This effect is however, yet to be demonstrated *in vitro *studies.

In contrast another study reported that among hemoglobin E patients treated with artemisinin derivatives, the presence of hemoglobin E trait was associated with an estimated 2.9-fold increase in parasite clearance rate (95% CI, 1.4-6.3; *P = *.006) [6].

The present study looked at the role of G6PD deficiency in gametocyte carriage and found that the proportion of gametocytes carried was similar in G6PD-deficient and G6PD-normal individuals after treatment at day 3. Similar percentage of gametocyte carriage has also been shown with ACT in the general population in Mali [13,28].

The clearance of *P. falciparum *using ACT in G6PD-deficient patients with uncomplicated malaria may require both enzymatic assay and genetic analysis of the mutation to detect and characterize G6PD deficiency. In clinical trials, whilst enzymatic assays seem to more closely approximate biologic function and correlate with protection, genetic analysis may uncover novel variants causing disease and thereby provide important insights for treatment of uncomplicated malaria.

The likely explanation for a lack of difference in parasite clearance time between those with and without G6PD deficiency is simply that clearance was rapid in all groups due to the rapid activity of artemisinin derivative, and that, if there was any difference, it could not be discriminated with once-daily assessment.

## Conclusions

The study findings suggest that G6PD deficiency did not increase or reduce the parasite clearance and the efficacy of ACT. The biological assays that detect G6PD enzyme activity during malaria treatment may reflect the level of G6PD deficiency and, therefore, may more accurately assess parasite clearance in the presence of treatment rather than using PCR methodology to determine G6PD deficiency as in this study. Other confounding factors such as other host erythrocyte variants and host relative immunity may also play a crucial role in G6PD activity. An *in vitro *study and *in vivo *with several daily assessments may be help to understand the interaction between parasite clearance, ACT and G6PD deficiency.

## Competing interests

The authors declare that they have no competing interests.

## Authors' contributions

IS, MAT, AD, AKK, AW, JK-M and OKD participated in study design. IS, AKK and MTA contributed in data management and analysis. AKK, SD, and AG did the molecular analysis in determining the status of G6PD deficiency. All authors participated in the preparation of the manuscript and approved the final version.
